# Field-based insights to the evolution of specialization: plasticity and fitness across habitats in a specialist/generalist species pair

**DOI:** 10.1002/ece3.202

**Published:** 2012-04

**Authors:** Timothy Griffith, Sonia E Sultan

**Affiliations:** 1 Department of Biology, Georgetown CollegeGeorgetown, KY 40324; 2 Department of Biology, Wesleyan UniversityMiddletown, CT 06459

**Keywords:** Generalization, niche breadth, phenotypic plasticity, shade tolerance, specialization

## Abstract

Factors promoting the evolution of specialists versus generalists have been little studied in ecological context. In a large-scale comparative field experiment, we studied genotypes from naturally evolved populations of a closely related generalist/specialist species pair (*Polygonum persicaria* and *P. hydropiper*), reciprocally transplanting replicates of multiple lines into open and partially shaded sites where the species naturally co-occur. We measured relative fitness, individual plasticity, herbivory, and genetic variance expressed in the contrasting light habitats at both low and high densities. Fitness data confirmed that the putative specialist out-performed the generalist in only one environment, the favorable full sun/low-density environment to which it is largely restricted in nature, while the generalist had higher lifetime reproduction in both canopy and dense neighbor shade. The generalist, *P. persicaria*, also expressed greater adaptive plasticity for biomass allocation and leaf size in shaded conditions than the specialist. We found no evidence that the ecological specialization of *P. hydropiper* reflects either genetically based fitness trade-offs or maintenance costs of plasticity, two types of genetic constraint often invoked to prevent the evolution of broadly adaptive genotypes. However, the patterns of fitness variance and herbivore damage revealed how release from herbivory in a new range can cause an introduced species to evolve as a specialist in that range, a surprising finding with important implications for invasion biology. Patterns of fitness variance between and within sites are also consistent with a possible role for the process of mutation accumulation (in this case, mutations affecting shade-expressed phenotypes) in the evolution and/or maintenance of specialization in *P. hydropiper*.

## Introduction

Species differences in ecological amplitude pose a conundrum for evolutionary ecologists. While some generalist species occur in a wide array of habitats or host types, many, often related, species are ecological specialists, found only in a narrower subset of conditions (reviewed in Janzen 1978; Strong et al. 1984; Jaenike 1990; Berenbaum 1996). Closely related species can also differ in their degree of adaptive plasticity (Sultan 2003), which may influence their ability to persist in different habitats. If the ability to occupy and reproduce in a wide array of habitats is beneficial, why are species with relatively narrow ecological distributions so prevalent in many clades (Bernays and Graham 1988; Futuyma and Moreno 1988; Via 1990; Holt and Gaines 1992; Fry 1996; Kawecki et al. 1997; van Tienderen 1997; Klassen 2002)? Do these “specialists” show superior function and fitness in these conditions as the term implies, or are they simply restricted to those conditions by poor performance in other habitats, due to genetic constraints on plasticity and/or demographic processes? A consensus on these key questions has proved elusive, despite substantial interest and an extensive empirical literature (Berenbaum 1996; Fry 2003; Stireman 2005). While experimental studies have greatly clarified the underlying issues, data on realized plasticity and fitness in natural field contexts are scarce. Such data can provide essential insights to both the mechanisms underlying ecological amplitude, and the potential constraints on its evolution. Here we present a comparative field study in naturally evolved populations of a closely related generalist/specialist species pair, across a well-understood aspect of habitat variation.

*Polygonum persicaria* L. is an ecological generalist that grows in a wide range of light habitats from full sun to moderate canopy shade, while *P. hydropiper* L. is a more restricted or “specialized” species found predominantly in full sun and only occasionally in partial shade (Sultan et al. 1998). With increasing density, shade from neighboring plants reduces the quantity of light energy received and changes light quality by altering the ratio of red:far red light habitats (Smith 1982, 1994). Canopy shade also changes light quality and quantity, but the reduction in light quantity can often be far more severe than under neighbor shade (Smith 1982; Donohue et al. 2000). Together, neighbor and canopy shade can create a mosaic of habitats that strongly affect plant growth and reproduction (Donohue et al. 2000; Dorn et al. 2000), such that fitness across these light habitats constitutes a critical aspect of plant ecological breadth.

Adaptive phenotypic plasticity in response to variation in light conditions is extremely well studied (Hutchings and de Kroon 1994; Aphalo and Ballaré 1995; Stuefer and Huber 1998; Dorn et al. 2000; Callahan and Pigliucci 2002; Sleeman et al. 2002; Schmitt et al. 2003). Larger leaves and/or increased allocation to leaf tissue are often observed in shaded individuals, plastic responses which maximize light capture (Sultan and Bazzaz 1993; Bazzaz 1996; Fitter and Hay 2002; Sultan 2003; Griffith and Sultan 2006). Plants also require more time to allocate carbon and other nutrients to developing offspring in light-limited and other stressful environments (Stanton et al. 2000), so that earlier flowering and the maintenance of reproductive allocation is highly adaptive in shaded environments (Morgan and Smith 1979; Donohue et al. 2000; Dorn et al. 2000; Stanton et al. 2000; reviewed in Donohue 2003).

Several constraints may limit the evolution of an adaptively plastic, generalist species. One possible constraint is inherent performance trade-offs that occur if alleles and associated traits that increase fitness in one environment reduce success in others (Levins 1968; Taper and Case 1985; Lynch and Gabriel 1987; Via 1990; Wilson and Yoshimura 1994; Roff 1997; Fry 2003). The presence of genetically based adaptive trade-offs would be confirmed at the population level by local adaptation to habitat (e.g., Caillaud and Via 2000), and at the genotype level by negative genetic fitness correlations between habitats (Via 1991; Wilson and Yoshimura 1994). A second possible explanation for the evolution of specialists is the existence of specific constraints on the evolution of adaptive phenotypic plasticity in certain taxa (van Tienderen 1991; Sultan 1992; Spitze and Sadler 1996; van Tienderen 1997). Adaptive plasticity in ecologically important traits can increase environmental tolerance and thus the range of habitats in which a species maintains high fitness (Williams et al. 1995; Bazzaz 1996; Lortie and Aarssen 1996; Weinig 2000; Sultan 2001; Parker et al. 2003; Voesenek et al. 2004). However, genetic maintenance costs associated with the ability to produce multiple, adaptive phenotypes (DeWitt et al. 1998; Scheiner and Berrigan 1998; reviewed in Agrawal 2001; Berrigan and Scheiner 2004) could constrain the evolution of such plastic generalists (van Tienderen 1991; Scheiner 1993; Sultan and Spencer 2002).

A third possible explanation for the evolution of specialization is based not on inherent genetic constraints, but rather on the accumulation of mutations that have deleterious effects in particular habitats (Fry 1996; Kawecki et al. 1997; Kawecki and Abrams 1999). When different habitats in a species’ range contribute unequal numbers of offspring to the next generation due to differences in either habitat productivity, frequency, or population size, mutations that reduce fitness in habitats contributing less to the next generation will be more slowly eliminated by selection than mutations that reduce fitness in habitats with a greater offspring contribution (Kawecki et al. 1997). As disadvantageous mutations accumulate in the less productive or common habitats, average fitness in these habitats will steadily erode (Fry 1996; Kawecki et al. 1997; Zeyl et al. 2001), and specialization (i.e., ecological restriction) to the more common or productive habitats will evolve as the result of this fitness loss. Supporting evidence for this process can be sought in intraspecific patterns. Specialist species should have high genetic variance for fitness in less productive or common habitats where deleterious mutations are predicted to accumulate (Fry 1996), and low genetic variance in more productive habitats where beneficial mutations are rapidly fixed (Whitlock 1996).

Unlike most existing studies of ecological specialization, our results provide comparative data at the species, population, and genotype levels. Such data are needed to evaluate the potential for genetically mediated fitness trade-offs and constraints on the evolution of plastic generalists (Futuyma and Moreno 1988; Sultan 1992; van Tienderen 1997). We performed a field reciprocal transplant of the two *Polygonum* species into two sites (full sun and partial canopy shade) where both naturally occur, planting individuals at two densities within each site to create the range of densities in which these plants naturally grow. With this design, we first tested whether the species’ differences in lifetime reproductive output in the four site and density combinations supported their characterization respectively as a generalist and a specialist. Then, we used these data to evaluate the two major hypotheses for the evolution of specialization in an ecological context, by comparing realized fitness in natural habitats at the species, population, and genotype levels. Specifically we (a) looked for patterns of fitness trade-offs at the population and genotype levels and (b) measured plasticity costs in each species. We also compared species’ differences in genetic as well as environmental variance for fitness in site and density treatments. Measured levels of naturally occurring herbivory provided an important context for interpreting these comparisons.

## Methods

### Study species

*Polygonum persicaria* and *P. hydropiper* (Polygonaceae) are closely related colonizing species (=*Persicaria maculata* and *P. hydropiper*; Kim and Donaghue 2005) introduced to North America over 300 generations ago from Eurasia (Gleason and Cronquist 1991). The species share similar life histories as obligately annual herbs with mixed breeding systems and indeterminate reproduction, producing numerous single-seeded fruits (achenes) until plants are killed by autumn frost (references in Sultan et al. 1998). Within this region, these species differ in ecological breadth: *P. persicaria* occurs in full sun to moderately shaded habitats while *P. hydropiper* is largely confined to full sun (or rarely partially shaded) sites (Sultan et al. 1998). The species often co-occur in open, nutrient-rich sites (references in Griffith and Sultan 2006).

### Experimental sample

We collected achenes from sympatric populations of *P. persicaria* and *P. hydropiper* growing in two sites representing the range of light conditions in which these species co-occur. One pair of sympatric populations occurred along the edge of a grazed pasture partially shaded by trees (“shade site,” ca. 30% available midday photosynthetically active radiation at *Polygonum* canopy, Towle Paddock, Dover, MA, 42.2°N, 71.3°W, Sultan et al. 1998), while the second set of populations occurred in an open field exposed to full sun with a large area of contiguous *Polygonum* habitat (“full sun site,” 100% available photosynthetically active radiation at canopy, Mount Hermon Farm, Mount Hermon, MA, 42.7°N, 72.4°W, Sultan et al. 1998). Nutrient levels and spring/summer moisture conditions are very similar in these sites (Sultan et al. 1998). Plants from each population of each species were grown and allowed to self-fertilize for two generations in a uniform glasshouse environment to generate 10–13 inbred lines from each of the two populations per species and eliminate possible maternal environmental effects due to source site (details in Sultan 2001). Achenes from these inbred lines were stratified in distilled water at 4°C for 6 weeks, then sown (12–13 May 2003) into flats filled with Metro-mix 360™ (Scotts Company, http:\\www.scotts.com). Seedlings germinated in the glasshouse under natural day lengths at 22°C day/18°C night and were transplanted into the field (27–30 May) at the first true leaf stage. One seedling per line was randomly assigned to each of two density treatments in eight replicate blocks at the two sites (see next paragraph), for an experimental sample of 8 seedlings per line per treatment per site (8 plants per line × 46 lines (24 *P. persicaria*, 22 *P. hydropiper*) × 2 treatments × 2 sites; total *N*= 1472).

### Site and density treatments

Experimental plots divided into eight replicate blocks were established at the shade (Towle Paddock) and full sun (Mount Hermon Farm) sites from which the populations were collected. Seedlings were transplanted into low-density (30 cm apart) and high-density (5 cm apart) treatments in each block. These treatments were based on the range of densities in natural populations at these sites and thus comprised realistic competitive microsites. To minimize edge effects, each treatment plot was surrounded by a border row. In the low-density treatment, grass was manually clipped once per week to simulate sheep and horse grazing that occurred at these sites.

### Data collection

Reproductive onset (flowering time), leaf size, herbivore damage, and fitness components were recorded. Flowering status was recorded twice per week until all plants had flowered (only terminal flowering was recorded, although a small proportion of *P. hydropiper* flowers are produced axially). Leaves were not measured until all plants had flowered. At that point, the three most recently produced, fully formed intact leaves were collected from each plant, traced, and scanned in an optical leaf area meter (LI-3100, LICOR, Lincoln, NE, USA) to determine mean individual leaf area (MLA). During the growing season, many leaves were partially consumed by insect herbivores, primarily Japanese Beetles (*Popillia japonica*). Leaf consumption was estimated (28 July–7 August) using a log scale to categorize the percent herbivory of the five most recently formed leaves (0 = 0–3%, 1 = 3–6%, 2 = 6–12%, 3 = 12–25%, 4 = 25–50%, 5 = 50–100% consumed). Average leaf consumption was then computed for each plant.

All experimental plants were allowed to grow until they naturally senesced (>50% of the plant's leaves senescent) and then harvested (26 August–18 September). Total dry above-ground mass was measured for all plants, while total mass of achenes, leaves, and stems was measured separately for plants in two blocks per site (1 plant per line per population per species per treatment in each block). These plant mass components were used to calculate reproductive allocation (ratio of achene mass to total above-ground plant mass). Total reproductive output included achene mass at harvest and previous bi-weekly achene collections that minimized the loss of mature achenes over the course of the growing season. Correlations between above-ground dry mass and total achene mass were computed for each population in each site and density combination and used to estimate total lifetime reproductive output (fitness) for all experimental plants.

### Statistical analyses

ANOVA was used to identify species differences in trait expression and fitness across site and density conditions (JMP v. 5.0.1). Models included species, population (within species), site, density, and block (within site) as fixed main effects. All two- and three-way interactions between species, population within species, site, and density were included. For each trait, a post-hoc Tukey's HSD test was used to identify significant differences between species across all site and density combinations. We used reproductive output as a measure of absolute fitness within each treatment (Stanton and Thiede 2005). Reproductive output was log *x*+ 1 transformed (Sokal and Rohlf 1995).

A second set of ANOVAs was used to test for local adaptation by identifying population and population by site effects on reproductive output (fitness) within each species. These models included population, line (within population), site, density, and block (within site) as main effects, and all two- and three-way interactions between population, line (within population), site, and density. Line was treated as a random effect and terms were tested over an appropriate synthetic denominator (JMP v. 5.0.1). Block was treated as a fixed effect because blocks were chosen to encompass topographic and vegetational variation across the site rather than randomly. Transformations and post-hoc tests were conducted as described above.

To assess the magnitude of fitness trade-offs, we computed the across-environment genetic correlations for fitness between all possible combinations of environments (site and density conditions). For each pair of environments, genetic correlations were computed as the ratio of the variance between lines to the sum of variances between lines and the line × environment interaction (Yamada 1962; Fry 1992). This computational method reduces bias generated by outliers and thus provides a robust test for the presence of genetic trade-offs within a species (Rausher 1984). Variances were computed from ANOVA models that included environment, population, and line (nested within population) as main effects and all two-way interactions with environment (cf. DeWitt et al. 1998; Donohue et al. 2000). Fitness measures were again log *x*+ 1 transformed.

Finally, we computed the relative fitness of each genotype in each site and density treatment to determine whether some genotypes had consistently high or consistently low fitness across treatments. Such a pattern would indicate that fitness trade-offs do not preclude the evolution of high-fitness genotypes across a broad range of growing conditions. Relative fitness was computed as the reproductive output for each genotype divided by the mean reproductive output of all genotypes of that species in a particular site and density treatment.

To assess whether plastic changes were adaptive, we measured selection gradients (β) for each trait in each environment. We performed genotypic selection analyses to measure the adaptive value of trait variation within each site and density treatment (Lande and Arnold 1983; Rausher 1992). Genotype (line) means were computed for each trait to reduce the impact of microsite variation on the correlation between trait expression and fitness (Stinchcombe 2002). Lines from populations and species were pooled to increase the range of trait variation and thus better detect the strength and direction of selection (Wade and Kalisz 1990; Weinig 2000; Etterson 2004). Relative fitness was computed for each line within each site and density treatment as the ratio of mean total achene mass for that line to the mean total achene mass of each site and density combination. Linear selection gradients (β) were computed for the MLA, percent reproductive allocation, and flowering time to determine whether selection acted on each trait independent of the other. Selection gradients were computed as the partial regression coefficients of the standardized trait values on relative fitness. Species and block were included in the model to remove these effects from the computed selection gradients. Interactions between traits and species were not included in the final model because in almost all cases they were nonsignificant and did not affect the significance of the selection gradients.

We measured the costs of plasticity for traits exhibiting statistically significant plasticity in accordance with adaptive predictions. Multiple regression was used to measure costs of plasticity in each focal environment (site and density treatment) relative to each reference environment (Donohue et al. 2000; Dorn et al. 2000; Poulton and Winn 2002). For each focal environment, fitness was regressed against trait values and their plasticities (relative to a reference environment). Line (genotype) means were used for trait and fitness values. Because there was a significant effect of block on all trait and fitness values (see Table 1), the effect of block was removed in each environment by using a one-way ANOVA with trait as the dependent variable and block as the sole independent variable. Line means for traits were computed by adding the residuals from each ANOVA to the mean trait value in each environment. Line means for fitness were calculated using just the residuals from each ANOVA, and thus constitute a measure of relative fitness in each environment. Plasticity for each line was measured as the difference between mean trait values in the focal and reference environment. A significant negative partial regression coefficient for trait plasticity indicates that plasticity has a negative effect on fitness independent of the trait value *per se* in that environment (Dorn et al. 2000) and is generally interpreted as a maintenance cost of plasticity (DeWitt et al. 1998; Scheiner and Berrigan 1998).

**Table 1:** Selection gradients (β) for traits in each light and density treatment.

	MLA	%Repro allocation	Flowering time
Full sun HD	−0.033	0.098	0.538*
Full sun LD	0.073	0.010	0.641***
Shade HD	0.224*	0.512***	−0.102
Shade LD	0.025	0.064	1.151***

HD, high density; LD, low density; MLA, mean leaf area.

* *P* < 0.05;

** *P* < 0.01;

*** *P* < 0.001. Bold values significant at α= 0.05.

Variance components for fitness were calculated to estimate the percent of between-line variance (i.e., genetic variance) relative to total phenotypic variance (Donohue et al. 2005). This percentage is analogous to broad sense heritability and was here used to compare genetic variance expected in different sites and densities. Variance components were calculated separately for each site and density combination using ANOVA with population, line (within population), and block as main effects (JMP v. 5.0.1). Variance components were estimated using a least-squares analysis, which is robust to relatively small shifts in design balance (Fry 1992) as was the case with this study. To confirm that design shifts did not substantially influence estimates of variance components, we also estimated variance components using a restricted maximum likelihood analysis (REML algorithm JMP v. 5.0.1). Maximum likelihood variance component estimates differed by less than 1% from the least-squares estimates and so only the latter were reported. A significant line effect indicated the presence of genetic variation.

## Results

### Fitness and trait expression in contrasting sites

**Figure 1:**
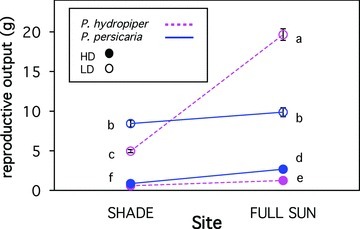
Fitness (total reproductive output) of *P. hydropiper* and *P. persicaria* at each site and density. Different letters indicate significant differences in a post-hoc Tukey's HSD test of all possible site and density combinations. Error bars ± 1 SE.

Species differences in fitness (measured as total lifetime reproductive output) were affected by both site and density (Table 2, species × site, density, and site × density terms). The generalist species, *P. persicaria*, had greater reproductive output than its congener in three of the four site and density combinations (Fig. 1): full sun/high density, shade/low density, and shade/high density (although this difference was not significant in a post-hoc Tukey's HSD test). However, the fitness difference was reversed in the most productive conditions—full sun/low density—where the reproductive output of the specialist species, *P. hydropiper*, was 2.0 times that of *P. persicaria* (Fig. 1).

**Table 2:** ANOVA *F* statistics for reproductive output (fitness) and allocation, leaf size, and phenology.

	Reproductive output	%Repro allocation	MLA	Flowering time
Sp	0.1	112.0***	2.5	20331.8***
Sp × Site	106.9***	29.5***	19.6***	7.9**
Sp × Density	57.3***	12.4***	14.8***	12.0***
Sp × Site × Density	165.0***	0.6	17.2***	7.1**
Pop(Sp)	78.4***	6.2**	16.1***	106.1***
Pop(Sp) × Site	11.8***	1.5	2.7	5.9**
Pop(Sp) × Density	3.0	1.5	5.0**	1.0
Pop(Sp) × Site x Density	4.4*	0.1	3.2*	1.6
Site	407.0***	0.2	453.5***	4.9*
Density	3807.1***	40.5***	291.1***	17.1***
Site × Density	12.8***	7.3**	31.4***	5.5*
Block(Site)	27.6***	13.7***	45.5***	4.5***

* *P* < 0.05;

** *P* < 0.01;

*** *P* < 0.001. Bold values significant at α= 0.05 after sequential Bonferroni correction.

For both species, the adaptive value of trait responses shifted substantially between sites and density treatments. In the full sun site and the low-density treatment of the shaded site, there was significant selection for later flowering times but no significant selection on other traits (Table 1). By contrast, in the high-density plots of the shaded sites, there was significant selection for larger MLA and higher reproductive allocation, but no significant selection on flowering time (Table 1).

**Figure 2:**
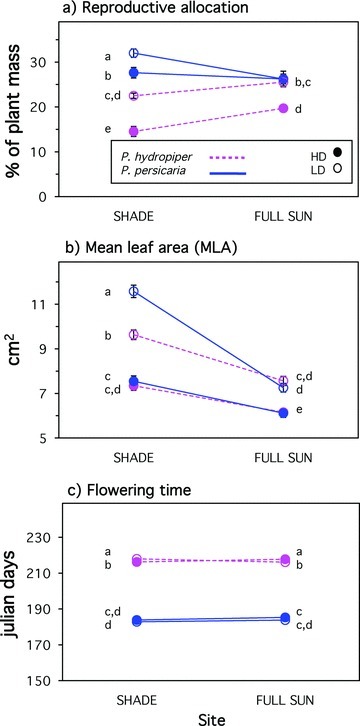
Trait expression at each site and density: a) reproductive allocation (percent of above-ground biomass); b) mean leaf area; c) first flowering date. Different letters indicate significant differences in a post-hoc Tukey's HSD test of all possible site and density combinations. Error bars ± 1 SE.

The species exhibited significant and large differences in their plastic responses to open versus shaded sites with respect to reproductive allocation and mean leaf area (MLA) (Table 2, species × site effect). Moreover, these changes corresponded with the shift to selection for greater reproductive allocation and higher MLA in the high-density shaded treatment. In the shaded site, the generalist *P. persicaria* increased the percent of above-ground biomass allocated to reproduction in both density treatments, while the specialist *P. hydropiper* was unable to maintain high reproductive allocation at either density (Fig. 2a). The increased reproductive allocation of *P. persicaria* at the shaded site resulted from reduced allocation to stem mass, which decreased by 10.1% and 6.7%, respectively, in the low-density and high-density treatments. By comparison, *P. hydropiper* reduced stem mass by only 3.5% and 1.2% in these treatments. *P. persicaria* also increased MLA more than *P. hydropiper* at the shaded site, especially at low density (Fig. 2b) where *P. persicaria* plants increased MLA by 60% compared with only 27% in *P. hydropiper*.

In contrast to reproductive allocation and leaf size, each species’ flowering time was consistent across light treatments but differed strongly between species. While flowering time differences between site and density treatments did not exceed 3 days for either species, *P. persicaria* flowered an average of 33.1 days earlier than *P. hydropiper* in all conditions.

### Fitness trade-offs: population differentiation and genetic fitness correlations

**Figure 3:**
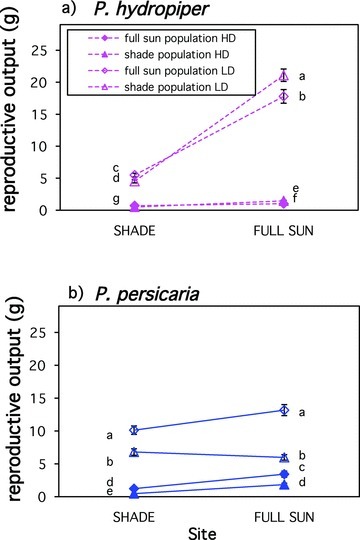
Population differences in fitness (total reproductive output) in a) *P. hydropiper* and b) *P. persicaria*. Different letters indicate significant differences in a post-hoc Tukey's HSD test of all possible site and density combinations. Error bars ± 1 SE.

Despite significant fitness differences between populations within each species (Table 2, pop(sp) and pop(sp) × site terms), native (home-site) populations did not generally have the highest fitness at each site. In *P. persicaria*, the full sun source population had significantly higher reproductive output than the shade source population at both sites and densities (Fig. 3b). In *P. hydropiper*, the native populations actually had lower fitness at each site in low-density conditions and in the full sun site at high-density conditions; there were no significant differences between source populations in the shaded/high-density treatment (Fig. 3a).

**Figure 4:**
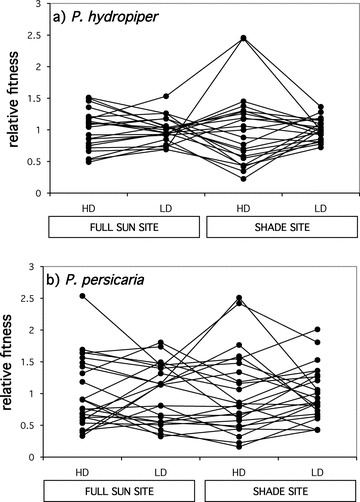
Fitness reaction norms for a) *P. hydropiper* genotypes and b) *P. persicaria* genotypes in each of four site and density environments (HD – high density, LD – low density). Relative fitness was computed in each treatment as the achene mass of the line divided by the average achene mass of all lines.

We also found no evidence in either species for fitness trade-offs at the genotype level. There were no significant negative genetic correlations for fitness across environments (Table 3): of the six possible site and density combinations for *P. persicaria*, genetic correlations for fitness were significant for only two (shade/low density vs. full sun/high density and shade/low density vs. full sun/low density), and both were positive. Similarly, genetic correlations in *P. hydropiper* were significant for only one site and density pair (full sun, low density vs. high density), and it too was positive. There were two negative genetic correlations in this species, but both were non-significant. Inspection of reaction norms confirmed that genotypes with high relative fitness in any one site and density treatment did not have consistently lower relative fitness in other site and density combinations (Fig. 4).

**Table 3:** Genetic correlation coefficients for fitness expressed in all site and density combinations. *P. hydropiper* coefficients are shown above the diagonal, *P. persicaria* coefficients below the diagonal.

	Full sun HD	Full sun LD	Shade HD	Shade LD
Full sun HD	–	0.472*	0.654	−0.455
Full sun LD	0.594	–	0.666	−0.289
Shade HD	0.360	0.654	–	0.316
Shade LD	0.887*	0.732***	0.415	–

HD, high density; LD, low density. Bold values statistically significant:

* *P* < 0.05;

** *P <* 0.01;

*** *P* < 0.001.

### Costs of adaptive plastic responses

There were no significant costs of plasticity (i.e., negative partial regression coefficients for the effect of plasticity on fitness) in the specialist species, *P. hydropiper*, for the two plastic traits (reproductive allocation and leaf area; Table 4A). In the generalist species, *P. persicaria*, there was a significant plasticity cost for reproductive allocation in the shade/high-density treatment with respect to trait expression in the full sun/low-density treatment (Table 4B). Furthermore, regardless of statistical significance, plasticity costs associated with light habitat were relatively low for both species (−0.038 ≤*r*≤−0.45) within each density treatment.

**Table 4:** Partial regression coefficients for trait plasticity on fitness (total reproductive output) indicating costs of plasticity (negative coefficients) in each focal environment with respect to all combinations of reference environments. (A) *P. hydropiper*, (B) *P. persicaria*.

(A)	Focal environment			
Reference environment	Full sun HD	Full sun LD	Shade HD	Shade LD
Full sun HD				
MLA	–	0.505	−0.321	0.705
%Repro	–	−0.300	−0.038	0.147
Full sun LD				
MLA	0.134	–	−0.503	0.067
%Repro	0.084	–	1.129	−0.121
Shade HD				
MLA	−0.426	−0.145	–	−0.184
%Repro	−0.152	0.178	–	0.262
Shade LD				
MLA	−0.180	−0.170	−0.803	–
%Repro	−0.200	0.250	0.201	–

(B)	Focal environment			
Reference environment	Full sun HD	Full sun LD	Shade HD	Shade LD

Full sun HD				
MLA	–	0.046	−0.046	−0.299
%Repro	–	−0.112	−0.246	−0.245
Full sun LD				
MLA	0.273	–	−0.170	−0.447
%Repro	−0.200	–	−0.567**	−0.544
Shade HD				
MLA	−0.051	−0.024	–	0.147
%Repro	−0.216	−0.022	–	0.023
Shade LD				
MLA	−0.010	−0.080	0.127	–
%Repro	0.041	0.068	−0.015	–

* *P* < 0.05;

** *P* < 0.01;

*** *P* < 0.001. Bold values significant at α= 0.05 after trait-wise sequential Bonferroni correction.

### Environmental and genetic fitness variance within species

*P. hydropiper* exhibited larger fitness differences between sites than did its generalist congener (Fig. 1). In *P. hydropiper*, absolute fitness (total reproductive output) was 297% greater in full sun than in shade in the low-density treatment, and 114% higher in the high-density treatment. By contrast, for *P. persicaria*, the absolute fitness differences between sites were only 17% at low density and 211% at high density. Thus, the average fitness difference between full sun and shade sites in *P. hydropiper* was 206% but only about half of that (114%) in *P. persicaria*.

Both species had significant among-line (genetic) variance in fitness, but only in certain site and density combinations (Table 5). Interestingly, there was no overlap between species in the conditions in which among-line variation was significant. In *P. hydropiper*, genetic variance was significant in the shade, high-density treatment, and in *P. persicaria*, genetic variance was significant in both the full sun and shade, low-density treatments. For *P. hydropiper*, the significant genetic variation found in the shade, high-density treatment was primarily the result of 2 lines with substantially higher fitness in this treatment (Fig. 4a). By contrast, in *P. persicaria* significant genetic variation reflected more widely dispersed genotypic fitnesses (Fig. 4b).

**Table 5:** Between-line variance as a percent of variance within and between lines for each species in each site and density treatment.

	*P. hydropiper*	*P. persicaria*
Full sun HD	5.0%	7.6%
Full sun LD	8.4%	11.7%*
Shade HD	23.5%***	7.3%
Shade LD	7.2%	23.5%***

Bold values statistically significant:

* *P* < 0.05;

** *P* < 0.01;

*** *P* < 0.001.

### Leaf herbivory

**Figure 5:**
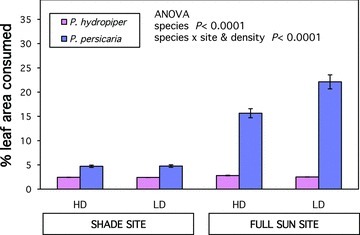
Insect herbivory (average percent of leaf area consumed) for both species at each site and density treatment. Error bars ± 1 SE. Significance tests are given based on a two-way ANOVA for species and site/density effects.

**Figure 6:**
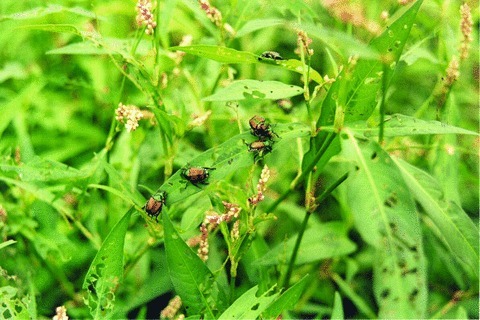
Herbivory by insects such as *Popilia japonica* causes substantial leaf damage to plants of *Polygonum persicaria*, as shown here at the MHF full-sun field plot.

By the middle of July, insects, primarily Japanese beetles (*Popillia japonica*), began consuming the leaves of plants at both sites. However, the amount of herbivore damage varied significantly by species and site (Fig. 5). Less than 3% of the leaf area of *P. hydropiper* plants was consumed by insect herbivores in either site. By contrast, for *P. persicaria*, 5% of leaf area was consumed by insects at the shade site while 16% and 22%, respectively, of leaf area was consumed in the high-density and low-density treatments of the full sun site (Fig. 6).

## Discussion

Our field experimental data confirmed that *P. hydropiper* is an ecological specialist that achieves very high fitness in one particular environment (high light, low density), while *P. persicaria* is a generalist species that maintains fitness across a wide range of canopy and density conditions. Genotypes of this generalist species also expressed greater adaptive plasticity for allocation and leaf size under shade conditions; in addition, their consistently early reproductive onset may promote success in diverse habitats. We found no evidence that the ecological narrowness of *P. hydropiper* resulted from commonly suggested genetic constraints: there were neither significant fitness trade-offs between light habitats, nor maintenance costs of adaptive plasticity, that would inherently limit the range of conditions in which the species’ genotypes could successfully grow and reproduce. However, the pattern of fitness variance in low- versus high-productivity sites suggests that in this system, herbivory and possibly metapopulation dynamics may influence the evolution and/or maintenance of specialist versus generalist ecological amplitudes. The key role of field herbivory levels on fitness in alternative environments underscores the importance of including ecological context in studies of evolutionary processes.

### Evolution of specialization versus generalization

#### Fitness trade-offs

Genetically based fitness trade-offs have often been evoked to explain the evolution of specialization: a genotype with highly adaptive expression in one environment will be less adaptive in others, leading to the evolution of species with high relative fitness in only a narrow environmental range (reviewed in Futuyma and Moreno 1988; Via 1990; Fry 1996, 2003). However, neither the generalist, *P. persicaria*, nor the specialist, *P. hydropiper*, exhibited such trade-offs. In both species, genotypes that achieved high relative fitness in particular site and density treatments were also able to achieve high relative fitness in other environments (thus providing the opportunity for selection to increase the frequency of high-performing genotypes across a broad range of light and density conditions). Indeed, the only significant genetic fitness correlations between site and density treatments were positive rather than negative, indicating that certain genotypes could achieve high fitness across an array of light conditions. A similar lack of negative genetic correlations has been observed in other studies in a range of taxa (examples in Futuyma and Moreno 1988; Fry 1996; Whitlock 1996).

It should be noted that the absence of significant negative genetic correlations does not absolutely preclude the existence of within-genotype fitness trade-offs: trade-offs could be undetectable due to a limited genetic sample, or because they are masked by alleles with large fitness effects across multiple environments (Rausher 1988; Shaw et al. 1995; Fry 1996; Weinig and Schmitt 2004). However, based on the substantial genetic variation we sampled among these naturally evolved lines (24 lines per species), we conclude that if undetected genotypic fitness trade-offs do exist in these populations, they must be very weak. Moreover, the high relative fitness of certain full sun evolved genotypes of *P. hydropiper* in the shaded site and vice versa suggests that even if genetically based fitness trade-offs exist in this species, they are not large enough to constrain the evolution of broadly tolerant genotypes.

#### Adaptive plasticity and plasticity costs

To the extent that adaptive plasticity allows individuals of a species to tolerate, and thus persist in, a wide range of environments, maintenance costs of plasticity constitute another possible genetic constraint to the evolution of ecological breadth (van Tienderen 1991; Scheiner 1993; DeWitt et al. 1998; reviewed in Sultan and Spencer 2002; Berrigan and Scheiner 2004). Greater adaptive plasticity may indeed contribute to the broader light distribution of the generalist species, *P. persicaria*. Plants of this species increased leaf area under canopy shade more than those of the specialist, *P. hydropiper*, a developmental response that is adaptive in shade environments in this study and for other herbaceous plant species (Chapin et al. 1987; Sultan and Bazzaz 1993; Bazzaz 1996). (Recall that the species’ samples represent an equally broad selective history in both types of light habitat.) *P. persicaria* plants also maintained or increased reproductive allocation under limited light (where total biomass is reduced), while *P. hydropiper* plants were unable to maintain reproductive allocation under either canopy or neighbor shade. Plastic responses in other traits not measured in this study, as well as the consistently earlier onset of reproduction in this species, may further contribute to the ability of *P. persicaria* plants to achieve relatively high fitness across a range of light environments (see Sultan 2003, 2009). Adaptive plasticity in response to contrasting light conditions may explain a lack of genotypic fitness trade-offs in these taxa: through such plasticity a “jack-of-all-trades” may in fact succeed in several environments by expressing distinct functionally appropriate phenotypes in each one (Sultan 2004).

Although our results support the view that plasticity contributes to ecological breadth for light habitat in *P. persicaria*, we found no evidence that plasticity costs for light-related traits constrain the evolution of broader ecological amplitude in *P. hydropiper*. Costs of plasticity in this specialist species were neither large nor statistically significant (but see caveats in previous section regarding statistical power to detect costs in any finite genetic sample). The only significant plasticity cost occurred in the highly plastic generalist species, indicating that this cost had been selectively outweighed by the benefits of plasticity.

#### Fitness variation in low-productivity environments

*P. hydropiper* plants had dramatically higher levels of absolute fitness in open versus shaded sites, especially in low-density conditions where the average individual output was nearly four times that of individuals in the open, high-density plots. These pronounced between-habitat fecundity differences would act to prevent any mutations that were unfavorable under shaded conditions from being eliminated as quickly by natural selection as mutations that were deleterious in the full sun sites (Kawecki et al. 1997; Kawecki and Abrams 1999; Zeyl et al. 2001). This raises an intriguing possible scenario: the gradual accumulation of mutations that were deleterious only in shaded conditions could lower the average fitness of *P. hydropiper* in these habitats, leading to its increasing “specialization” or ecological restriction to full sun conditions. Because ours is a retrospective study of naturally selected populations (in contrast to a prospective artificial-selection experiment), our results cannot definitively address this issue. However, it is interesting to note that the pattern of genetic variance within environments in *P. hydropiper* is consistent with such a mutation-accumulation scenario, as the highest genetic variance for fitness occurred in the least productive habitat (canopy shade with high neighbor density) where selection to eliminate deleterious mutations would be least effective. (The lack of significant negative genetic fitness correlations between densities indicates that microsite selection was unlikely to be a factor in the maintenance of genetic variation within a site.)

Population genetic theory predicts that specialist species should have high genetic variance for fitness in less productive or less common habitats where deleterious mutations are allowed to accumulate (Fry 1996), and low genetic variance in more productive habitats where beneficial mutations are rapidly fixed due to strong selection (Whitlock 1996). Drift and selection experiments with microorganisms over hundreds of generations indicate that such mutation accumulation may be responsible for the evolution of narrow ecological amplitude in these systems (e.g., Reboud and Bell 1997; Zeyl et al. 2001; MacLean and Bell 2002), although to date similar experiments with plants have not yielded any support for this hypothesis (Chang and Shaw 2003; Kavanaugh and Shaw 2005). Although not conclusive, our study is the first to produce results consistent with this hypothesis in a field setting, by demonstrating that high fitness variance for naturally occurring genotypes of a specialist taxon is expressed in unproductive habitats. Further studies in natural systems are needed to illuminate the possible role of dynamic stochastic processes in the evolution of specialization versus generalization.

#### A role for herbivory in the evolution of ecological breadth?

Our field study revealed dramatic differences in herbivore resistance that raise the intriguing possibility that this ecological factor may have contributed to the evolution of specialization and generalization in this species pair. Because *P. hydropiper* is far less susceptible to herbivory than *P. persicaria*, it has a dramatic growth advantage in full sun conditions where insect herbivores are most abundant and active. This growth advantage substantially increases reproductive asymmetry between light habitats for plants of the specialist species. Hence, the evolution of herbivore resistance in *P. hydropiper* might have created conditions that would allow mutations expressed in shade to accumulate, thereby promoting the evolution of greater ecological specialization. In contrast, *P. persicaria* is susceptible to herbivory; indeed, adaptive plastic responses to neighbor shade may reduce herbivore resistance (Kurashige and Agrawal 2005). Paradoxically, then, lack of herbivore resistance in plastic species such as *P. persicaria* may promote the evolution of generalization: with only a slight productivity difference between sun and shade habitats due to high herbivory in open sites, selection will remove mutations that are deleterious in shaded habitats at close to the same rate, leading to the evolution of light-generalist genotypes. Phylogenies of several taxa now indicate that generalist species can be derived from specialist species (Schluter 2000; Nosil 2002; but see also Stireman 2005); our results suggest that loss of a trait such as herbivore resistance could contribute to such evolutionary trajectories.

### Broader implications

#### Evolution of plasticity

The cause-and-effect relationship between species’ adaptive phenotypic plasticity, ecological amplitude, and evolutionary change is one of the central issues invoked by the phenomenon of plasticity (Whitlock 1996; Schlichting and Pigliucci 1998; Agrawal 2001; Donohue 2003; West-Eberhard 2003; Sultan 2004). This relationship is often seen as a one-way process whereby individual adaptive plasticity determines a species’ distribution with respect to distinct sites and habitats, and consequently its pattern of evolutionary divergence or stasis (Moran 1992; van Tienderen 1997; Sultan and Spencer 2002; Donohue 2003; Schlichting 2004). By contrast, our results raise the possibility that the chain of causation may also work in the opposite direction: phenotypic differences between species could affect their respective ecological amplitude so as to determine the degree of adaptive plasticity that subsequently evolves. In the present case, herbivore resistance and consequent steep fitness differences in *P. hydropiper* may have promoted its specialization to full sun, low-density habitats. This specialization in turn could explain why *P. hydropiper* has not evolved the same degree of adaptive plasticity as *P. persicaria*: because the former species persists in only a narrow range of habitats, it does not encounter strong selection for adaptive response to contrasting conditions. In other words, the initial evolution of specialization can prevent a species from experiencing the environmental heterogeneity that selectively favors plastic genotypes.

#### Invasiveness and generalization

These results also have surprising implications for our understanding of species’ invasiveness and its relation to ecological generalization. Escape from herbivores or other predators (enemy release hypothesis or ERH) is often suggested to explain why some introduced species are far more aggressive competitors in new geographic regions than in their native ranges (Keane and Crawley 2002; Wolfe 2002; Maron et al. 2004; Joshi and Vrieling 2005). For the pair of introduced *Polygonum* species in this study, this “escape” hypothesis would predict that the herbivore-resistant species, *P. hydropiper*, would achieve a broader ecological amplitude and be more invasive in its North American range than *P. persicaria*, which is readily consumed by both insect herbivores and mammal grazers. Surprisingly, the opposite is true: *P. hydropiper* has a narrower ecological distribution and is less abundant as a weedy colonizing species than *P. persicaria* (Simmonds 1945; Timson 1966; Sultan et al. 1998). This may reflect the unexpected role of herbivore resistance in the evolution of ecological specialization in this system. If reproductive asymmetry caused by herbivore resistance can lead to the evolution of specialization, then over several generations, herbivore resistance may actually *decrease* a species’ ecological amplitude and invasiveness. Contrary to expectations, a lack of predation pressure on an introduced species could evolutionarily lock the species into the role of a specialist, making it less likely to spread as a general invasive.
